# Anti-cariogenic activity of mutanocyclin, a secondary metabolite of *Streptococcus mutans*, in mono- and multispecies biofilms

**DOI:** 10.1128/spectrum.00183-25

**Published:** 2025-06-25

**Authors:** Fangting Huang, Yang Zhou, Dongru Chen, Huancai Lin

**Affiliations:** 1 Hospital of Stomatology, Guanghua School of Stomatology, Sun Yat-Sen University 623167 , Guangzhou, China; 2 Guangdong Provincial Key Laboratory of Stomatology, Sun Yat-Sen University 26469 , Guangzhou, China; University of Manitoba, Winnipeg, Manitoba, Canada

**Keywords:** mutanocyclin, *Streptococcus mutans*, *Streptococcus gordonii*, *Streptococcus sanguinis*, multispecies biofilms

## Abstract

*Streptococcus mutans* (*S. mutans*), a principal cariogenic pathogen responsible for dental caries, alters the oral biofilm ecology through the production of various metabolites. One such metabolite, mutanocyclin (MUC), a recently identified secondary metabolite, may have implications for both caries development and oral microbiome interactions. This study aimed to investigate the properties of MUC and its effects on *S. mutans*, as well as other oral commensal streptococci in mono- and multispecies biofilms. Our findings revealed that MUC significantly inhibited *S. mutans* planktonic growth, biofilm formation, lactic acid production, water-insoluble glucan synthesis, and extracellular polysaccharide production. In contrast, at lower concentrations, MUC stimulated the growth of *Streptococcus gordonii* (*S. gordonii*) and *Streptococcus sanguinis* (*S. sanguinis*). Additionally, MUC reduced the abundance of *S. mutans* and enhanced the antagonistic activity of *S. gordonii* and *S. sanguinis* against *S. mutans* in multispecies biofilms by upregulating the expression of the H_2_O_2_-related gene *spxB* and stimulating H_2_O_2_ production. These results suggest that MUC has potential for anti-cariogenic activity against *S. mutans* and for modulating oral microbial communities within the cariogenic microenvironment, providing new therapeutic strategies for preventing dental caries.

**IMPORTANCE:**

Dental caries is a major global health concern, affecting millions and contributing to reduced quality of life and economic burdens. Our study investigated the properties of mutanocyclin (MUC), a recently discovered secondary metabolite produced by *S. mutans*. Our work sheds light on the role of MUC in modulating microbial communities in the oral cavity. We demonstrated that MUC influences the formation and cariogenicity of *S. mutans* biofilms and its interactions with other beneficial oral bacteria. This research enhances our understanding of newly discovered secondary metabolites of *S. mutans* and offers a potential novel strategy for managing the microbial imbalances that lead to caries.

## INTRODUCTION

Oral diseases constitute a significant global public health issue due to their high prevalence (1). The 2017 Global Burden of Disease Study identified permanent dental caries as the most prevalent of 328 diseases (2). Dental caries affect not only the hard tissues of the teeth but also lead to complications that may exacerbate or contribute to systemic conditions, significantly reducing quality of life and causing substantial economic challenges (3). Therefore, preventing dental caries is essential to reducing its global impact and improving public health outcomes.

Dental caries is a biofilm-associated disease, with its development closely linked to microbial dysbiosis within dental biofilms (4). The composition of the oral microbiome is shaped by the local environment, and alterations in these conditions can modify microbial interactions within the biofilm, thereby increasing the risk of caries (5). *Streptococcus mutans* (*S. mutans*), a primary cariogenic bacterium, can exploit environmental disruptions to modify the plaque microecology by producing various metabolites. This process enhances its competitive advantage and contributes to the initiation and development of dental caries (6, 7).

Secondary metabolites, such as the bacteriocins produced by *S. mutans*, are essential for bacterial physiological functions and the ecological stability of microbial communities. The absence or accumulation of specific metabolites can disrupt the microecology of bacterial biofilms, affecting the survival, development, and inter- and intra-species interactions of microorganisms (8, 9). Recent studies have demonstrated that approximately 15% of *S. mutans* carry gene clusters responsible for polyketides (PKs) biosynthesis and non-ribosomal peptides (NRPs), which are involved in the production of the newly discovered natural secondary metabolite, mutanocyclin (MUC) (10, 11).

**Fig 1:**
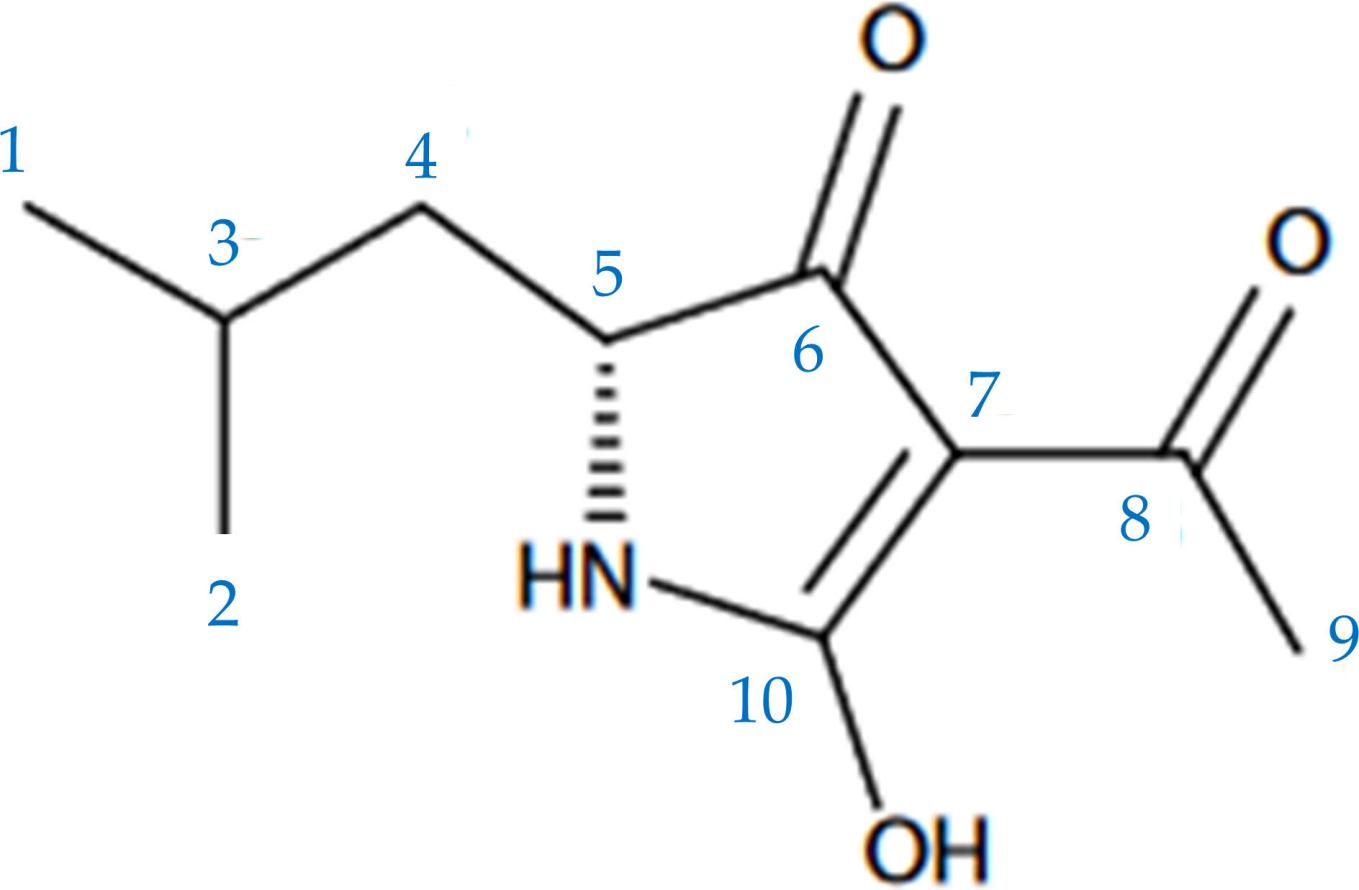
Chemical structure of MUC.

MUC was first found in 2019. Hao et al. (10) utilized *S. mutans* UA159 as a host to express biosynthetic gene clusters (BGCs) from anaerobic bacteria for secondary metabolite production. During this study, they identified MUC as a secondary metabolite derived from *BGC1*. MUC is closely associated with the adhesion and biofilm formation capabilities of *S. mutans*. MUC is a tetramic acid (Fig. 1) (10, 12–14). Unlike bacteriocins, it does not exhibit significant antibacterial activity against certain oral commensal bacteria under laboratory conditions (10). However, it exerted a more specific reduction in the relative abundance of *Limosilactobacillus fermentum* (*L. fermentum*) in an *in vitro* biofilm model derived from human saliva. Additionally, MUC has been shown to suppress filamentous growth and virulence of *Candida albicans* (*C. albicans*) by influencing the process of cell wall biogenesis and remodeling (15).

PKs/NRPs have been extensively studied in bacteria with large genomes, and the significance of these secondary metabolites is well established. However, research on PKs/NRPs in *S. mutans* is limited, particularly regarding relatively novel secondary metabolites. Among these, MUC has shown potential to influence the survival, development, and interactions of *S. mutans* with other bacterial species. Despite this, investigations into the role of MUC are in the early stages. The objective of our investigation was to preliminarily explore the characteristics of MUC and assess its impact on the biofilm development of *S. mutans* UA159 (a strain lacking *BGC1*) and other oral commensal streptococci. Furthermore, we sought to elucidate the mechanism underlying its activity. Identifying and characterizing this metabolite may provide new insights for preventing dental caries and identifying potential targets for modulating interactions between *S. mutans* and other commensal bacteria.

## RESULTS

### Cytotoxicity and antibacterial characteristics of MUC against planktonic *S. mutans*

**Fig 2:**
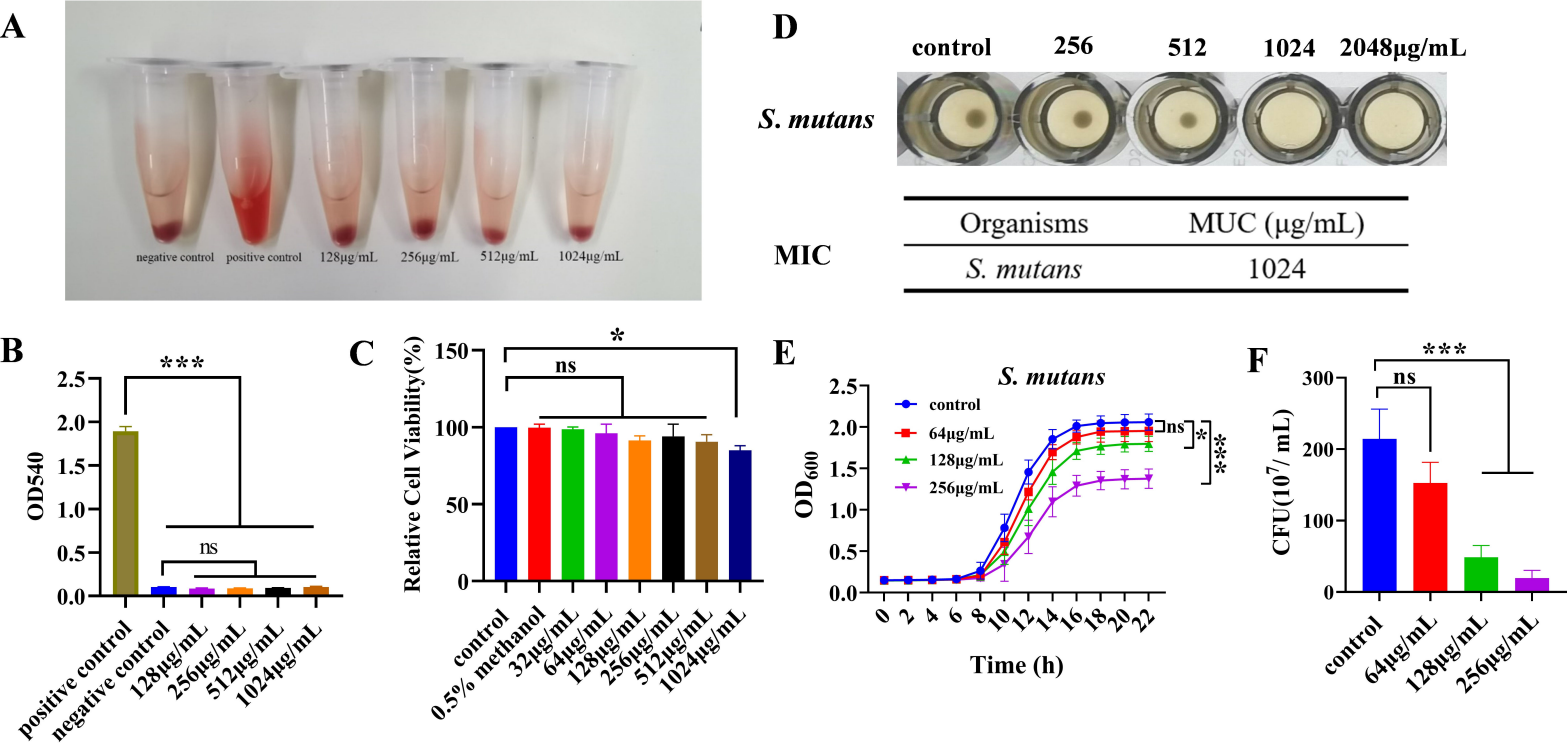
Biosafety and antibacterial characteristics of mutanocyclin (MUC). (**A**) Hemolytic assay. (**B**) Quantification of the hemolytic activity in the supernatants from the hemolysis assay. (**C**) Relative cell viability of human oral keratinocyte (HOK) cells in the cell cytotoxicity assay. (**D**) Minimum inhibitory concentration (MIC) of MUC against planktonic *Streptococcus mutans* (*S. mutans*). (**E**) Growth curves of *S. mutans* treated with various concentrations of MUC. (**F**) Colony-forming unit (CFU) counts of the planktonic *S. mutans* cultured for 24 h. *P < 0.05, ***P < 0.001, ns: no statistical significance.

The biosafety of MUC was evaluated by a hemolytic assay with erythrocytes and a cell cytotoxicity assay with human oral keratinocyte (HOK) cells. Results presented in Fig. 2A indicate that pronounced hemolysis of erythrocytes occurred in the positive control group, whereas no notable hemolysis was observed in the supernatant of the negative control group or the treatment groups. Quantitative analysis of supernatant samples from the hemolysis assay revealed that the optical density (OD) value of the positive control group was significantly higher. In contrast, no substantial differences were observed in OD values between the negative control group and the treatment group (Fig. 2B). Figure 2A and B confirm that MUC did not induce significant erythrocyte damage. Additionally, no significant variation in HOK cell viability was observed following exposure to MUC concentrations ranging from 32 to 512 µg/mL in the cytotoxicity assay. However, at a concentration of 1,024 µg/mL, MUC significantly reduced cell viability compared to the control group (Fig. 2C). Furthermore, a methanol concentration of 0.5% did not significantly influence the cell viability of HOK cells (Fig. 2C).

The antibacterial efficacy of MUC against *S. mutans* was assessed by determining the minimum inhibitory concentration (MIC), which was found to be 1,024 µg/mL (Fig. 2D). The growth curves demonstrated that MUC at doses of 128 and 256 µg/mL effectively suppressed the proliferation of *S. mutans* planktonic cells, whereas at a lower concentration of 64 µg/mL, the suppression was not significant (Fig. 2E). Quantitative colony counting following MUC (64–256 µg/mL) exposure revealed a dose-dependent reduction in the number of viable bacteria (Fig. 2F). And a methanol concentration of 0.5% (vol/vol) did not significantly influence the growth of planktonic *S. mutans* (Fig. S1). Our results indicated that MUC exhibited favorable biosafety and demonstrated effective antibacterial property against planktonic *S. mutans* at concentrations between 64 and 256 µg/mL.

### Inhibition of *S. mutans* biofilm formation, lactic acid production, and extracellular polysaccharide (EPS) accumulation by MUC

**Fig 3:**
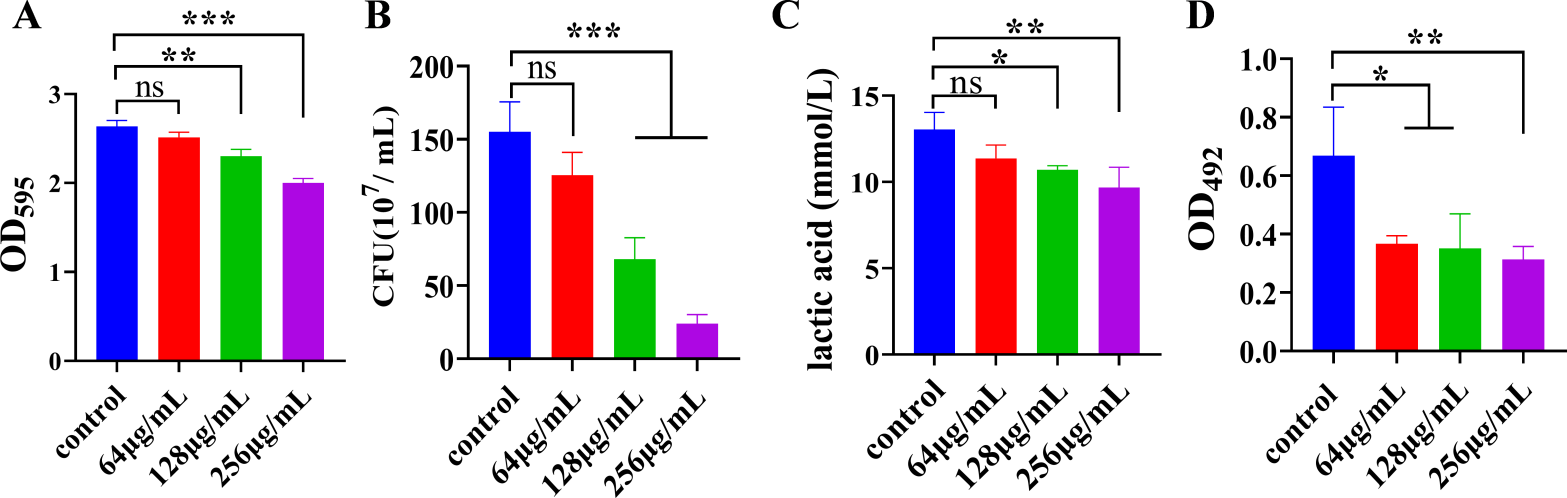
Effect of MUC on *S. mutans* biofilm formation, lactic acid production, and water-insoluble glucan (WIG) accumulation. (**A**) Effect of MUC on *S. mutans* biofilm formation. (**B**) CFU counts of *S. mutans* biofilms treated with various concentrations of MUC after 24 h of culture. (**C**) Lactic acid production within *S. mutans* biofilms. (**D**) Quantitative WIG detection within *S. mutans* biofilms. *P < 0.05, **P < 0.01, ***P < 0.001, ns: no statistical significance.

MUC’s impact on *S. mutans* biofilm formation was assessed using crystal violet staining. The findings indicated that MUC markedly decreased *S. mutans* biofilm development in a dose-dependent manner (Fig. 3A). Quantitative colony counts confirmed these results, showing a marked decrease in the number of viable bacteria following treatment with MUC (128 and 256 µg/mL) (Fig. 3B). To evaluate the effect of MUC on acid production, a primary cariogenic virulence factor of *S. mutans*, lactic acid levels were measured. Lactic acid production significantly decreased when MUC was administered at concentrations of 128 and 256 µg/mL compared to the control group (Fig. 3C). Furthermore, water-insoluble glucan (WIG) accumulation in the biofilm was measured using the phenol-sulfuric acid approach, revealing that MUC-treated groups produced significantly less WIG (Fig. 3D).

**Fig 4:**
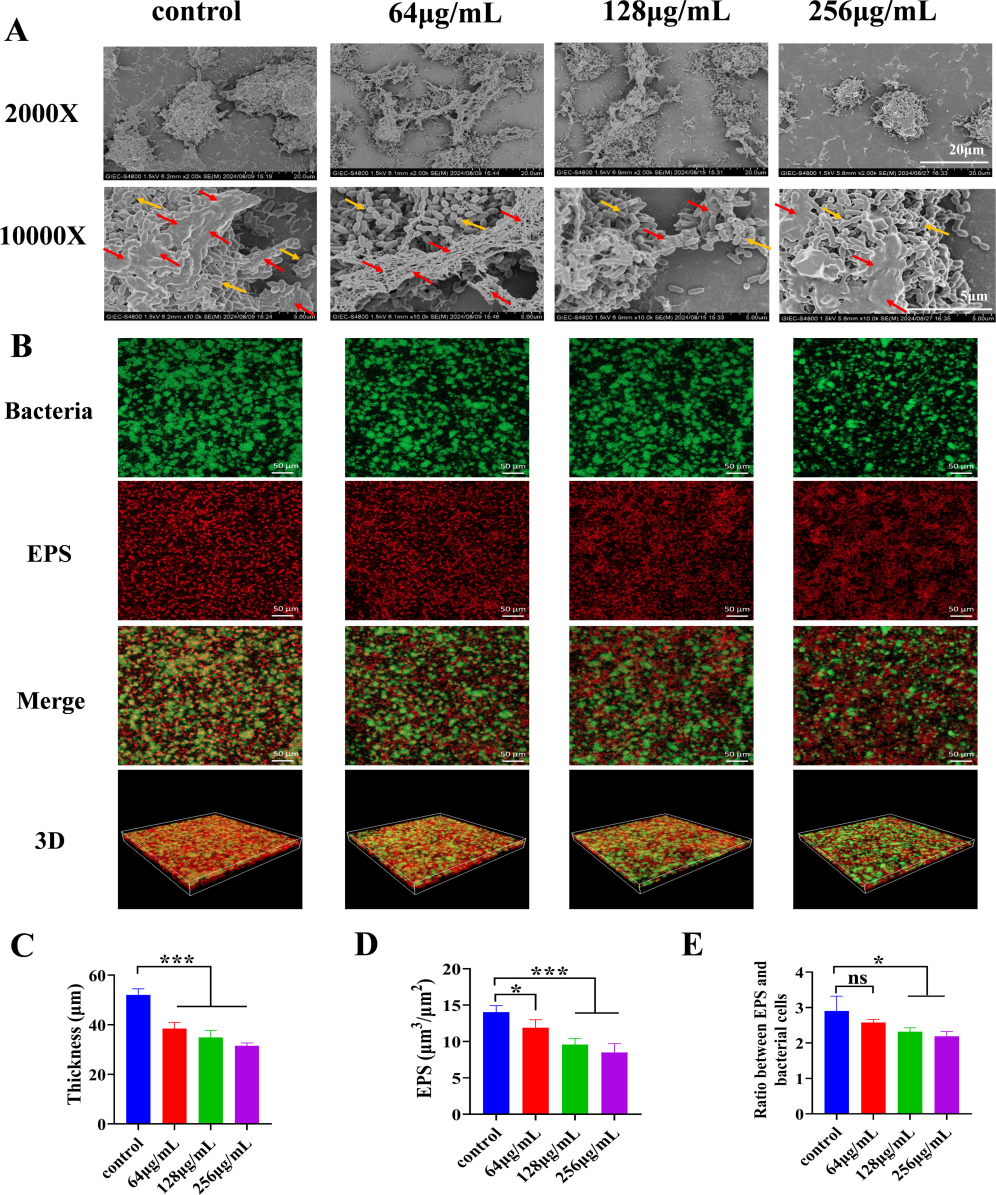
Effect of MUC on *S. mutans* biofilm morphology, thickness, and EPS accumulation. (**A**) Morphology of *S. mutans* biofilms treated with various concentrations of MUC, visualized by scanning electron microscopy (SEM) at 2,000× and 10,000× magnifications. (**B**) Representative confocal laser scanning microscopy (CLSM) images (20×) of *S. mutans* biofilms treated with various concentrations of MUC. Green fluorescence (SYTO9) represents live bacterial cells, while red fluorescence (dextran labeled with Alexa Fluor 647) represents EPS. (**C**) Quantification of biofilm thickness. (**D**) Quantification of EPS accumulation within biofilms. (**E**) Ratio of EPS to bacterial cell biomass. Red arrows indicate the extracellular matrix; yellow arrows indicate clustered microorganisms. *P < 0.05, ***P < 0.001, ns: no statistical significance.

The effect of MUC on biofilm morphology was analyzed using scanning electron microscopy (SEM). MUC treatment significantly inhibited the density of bacterial cells in *S. mutans* biofilms (Fig. 4A). Confocal laser scanning microscopy (CLSM) was employed to investigate the influence of MUC on biofilm architecture (Fig. 4B). The findings indicated that MUC-treated biofilms showed a significant reduction in thickness compared to the untreated control group, which corresponded with a significant decrease in EPS accumulation (Fig. 4C and D). Furthermore, the ratio of EPS to bacterial biomass decreased as the concentration of MUC increased.

Our results showed that MUC significantly inhibited *S. mutans* biofilm formation and cariogenic virulence, reducing bacterial counts, lactic acid production, biofilm thickness, WIG, and EPS accumulation. Furthermore, the ratio of EPS to live bacterial biomass was reduced (Fig. 4E). These results revealed MUC’s potential as a targeted approach to reduce *S. mutans* biofilm cariogenic virulence.

### Inhibition of bacterial viability and modulation of virulence-associated gene expression in *S. mutans* biofilms by MUC

**Fig 5:**
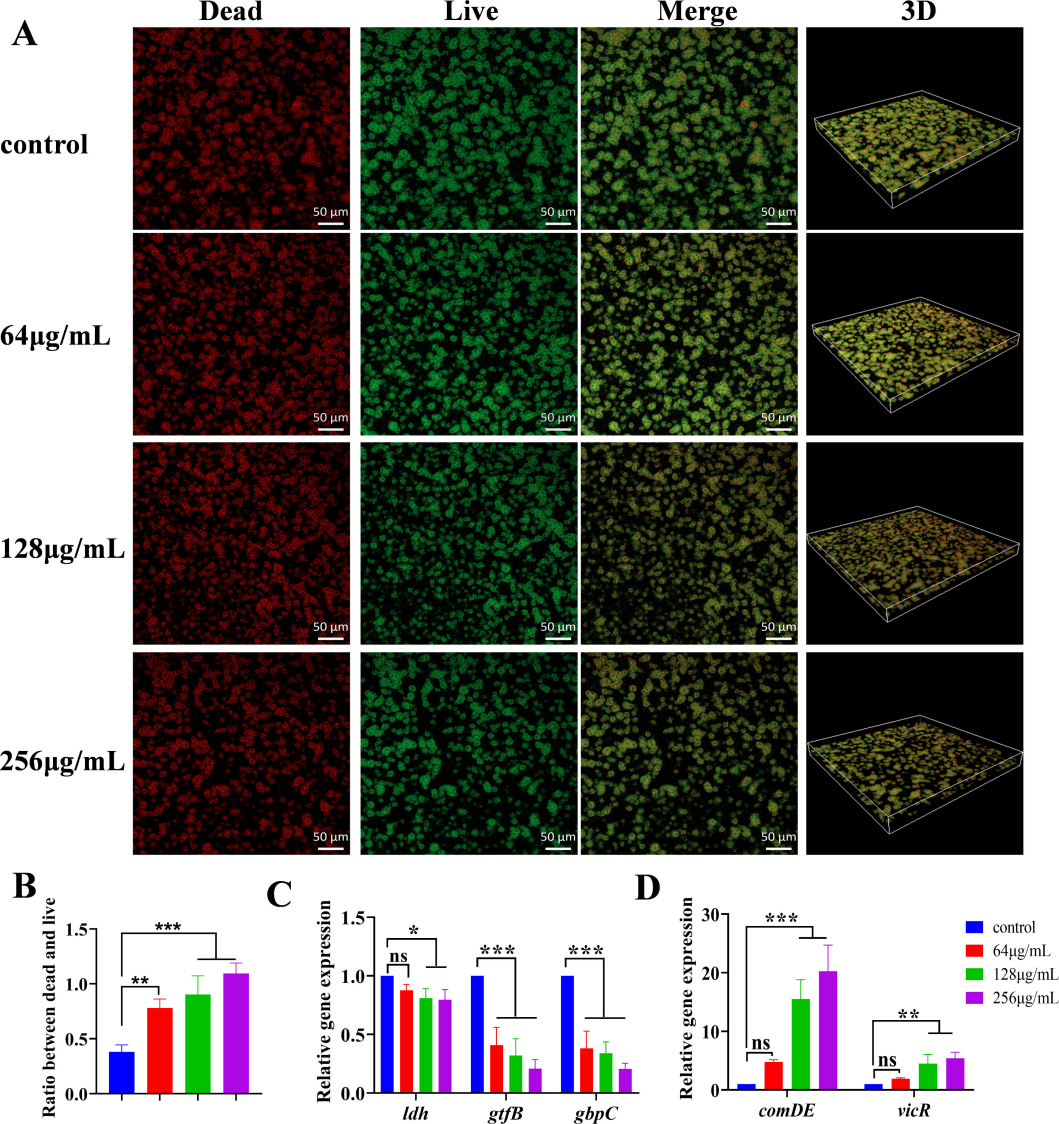
Impact of MUC on *S. mutans* biofilms bacterial viability and virulence-associated gene expression. (**A**) Representative CLSM images (20×) of live and dead bacteria within *S. mutans* biofilms. Red fluorescence (propidium iodide, PI) indicates dead bacteria, while green fluorescence (SYTO9) indicates live bacteria. (**B**) Ratio of dead to live bacteria within biofilms. (**C and D**) Quantification of mRNA expression levels of *ldh*, *gtfb*, *gbpC*, *comDE*, and *vicR* in *S. mutans* biofilms. *P < 0.05, **P < 0.01, ***P < 0.001, ns: no statistical significance.

Live/dead staining experiments were conducted to evaluate the effect of MUC on the bacterial viability of *S. mutans* biofilms (Fig. 5A). The MUC treatment led to a dose-dependent elevation in the ratio of dead to live bacteria (Fig. 5B). Quantitative reverse transcription polymerase chain reaction (qRT-PCR) was used to measure the expression levels of virulence genes linked to lactic acid production, biofilm formation, and stress responses. At concentrations of 128 and 256 µg/mL, MUC significantly inhibited the expression of the *ldh* gene. At a concentration of 64 µg/mL, the biofilm formation-associated genes *gtfB* and *gbpC* were downregulated by approximately 0.51-fold and 0.38-fold, respectively (Fig. 5C). MUC significantly reduced *gtfb* and *gbpC* expression in a dose-dependent manner. In contrast, the stress response-related gene *comDE* exhibited a 4.78-fold upregulation (Fig. 5D), while *vicR* expression increased by 1.88-fold when *S. mutans* biofilms were treated with 64 µg/mL MUC. Moreover, MUC significantly increased the expression of *comDE* and *vicR* in a dose-dependent manner. These findings indicated that MUC reduced *S. mutans* biofilm viability, enhanced bacterial stress responses, and diminished the pathogenicity associated with *S. mutans* lactic acid production and biofilm formation.

### Promotion of planktonic growth of *Streptococcus gordonii* (*S. gordonii*) and *Streptococcus sanguinis* (*S. sanguinis*) by low concentrations of MUC

**Fig 6:**
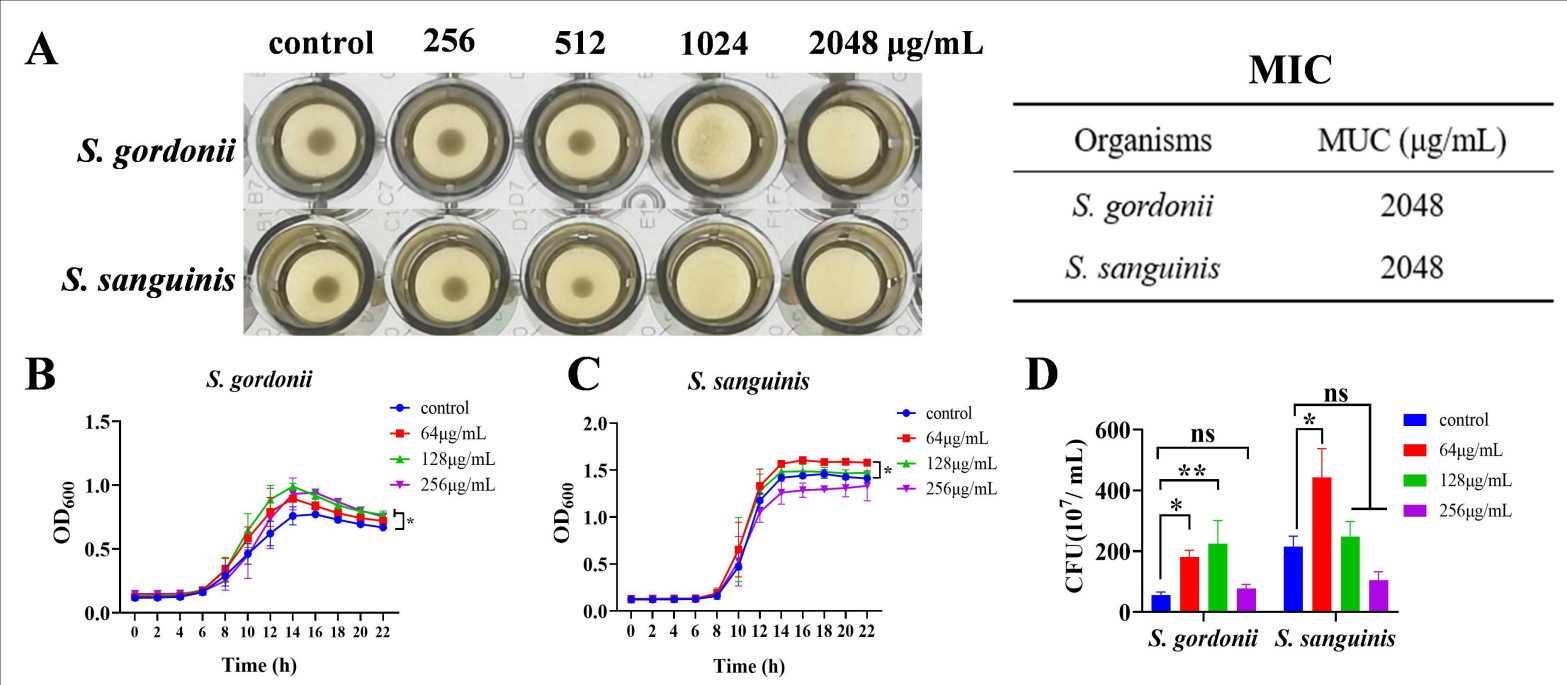
Effect of MUC on planktonic growth of *Streptococcus gordonii* (*S. gordonii*) and *Streptococcus sanguinis* (*S. sanguinis*). (**A**) MIC of MUC against planktonic *S. gordonii* and *S. sanguinis*. (**B and C**) Growth curves of *S. gordonii* and *S. sanguinis* treated with various concentrations of MUC. (**D**) CFU counts of *S. gordonii*, *S. sanguinis* after treatment with MUC and culture for 24 h. *P < 0.05, **P < 0.01, ns: no statistical significance.

The MIC of MUC against *S. gordonii* and *S. sanguinis* was determined to be 2,048 µg/mL (Fig. 6A). In contrast to its effect on *S. mutans*, MUC at concentrations of 128 and 256 µg/mL significantly promoted the proliferation of *S. gordonii* planktonic cells, whereas at 64 µg/mL, MUC had a non-significant effect on growth (Fig. 6B). For *S. sanguinis*, MUC promoted growth exclusively at 64 µg/mL, with the effect diminishing at higher concentrations (Fig. 6C). Quantitative colony counting demonstrated that MUC at 64 or 128 µg/mL promoted the proliferation of planktonic *S. gordonii* and *S. sanguinis* (Fig. 6D). These observations indicated that low concentrations of MUC promoted the planktonic growth of *S. gordonii* and *S. sanguinis* while inhibiting that of *S. mutans*. Therefore, MUC concentrations of 64 and 128 µg/mL were selected for subsequent experiments.

### Suppression of multispecies biofilm formation, lactic acid production, and EPS accumulation by low concentrations of MUC

**Fig 7:**
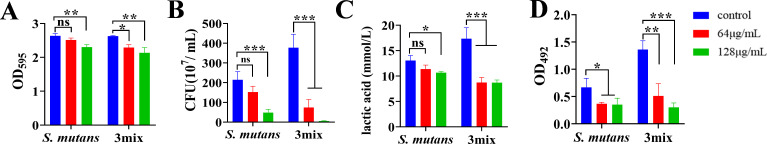
Effect of MUC on multispecies biofilm formation, lactic acid production, and WIG accumulation. (**A**) Effect of MUC on multispecies biofilm formation. (**B**) CFU counts of multispecies biofilm treated with various concentrations of MUC after culturing for 24 h. (**C**) Lactic acid production within multispecies biofilms. (**D**) Quantitative WIG analysis within multispecies biofilms.

Both the crystal violet staining assay and quantitative colony counting demonstrated that MUC significantly inhibited the formation of multispecies biofilms and decreased the number of culturable live bacteria within the biofilms (Fig. 7A and B). Treatment with MUC at 64 and 128 µg/mL also resulted in a significant reduction in lactic acid production and WIG accumulation compared to the control group (Fig. 7C and D). Moreover, the reduction in CFU counts, lactic acid production, and WIG accumulation was more pronounced in multispecies biofilms than in monospecies biofilms of *S. mutans* (Fig. 7A through D). Overall, MUC exhibited a significantly greater capacity to reduce cariogenic properties, including biofilm formation, lactic acid production, and WIG accumulation in multispecies biofilms relative to *S. mutans* monospecies biofilms.

**Fig 8:**
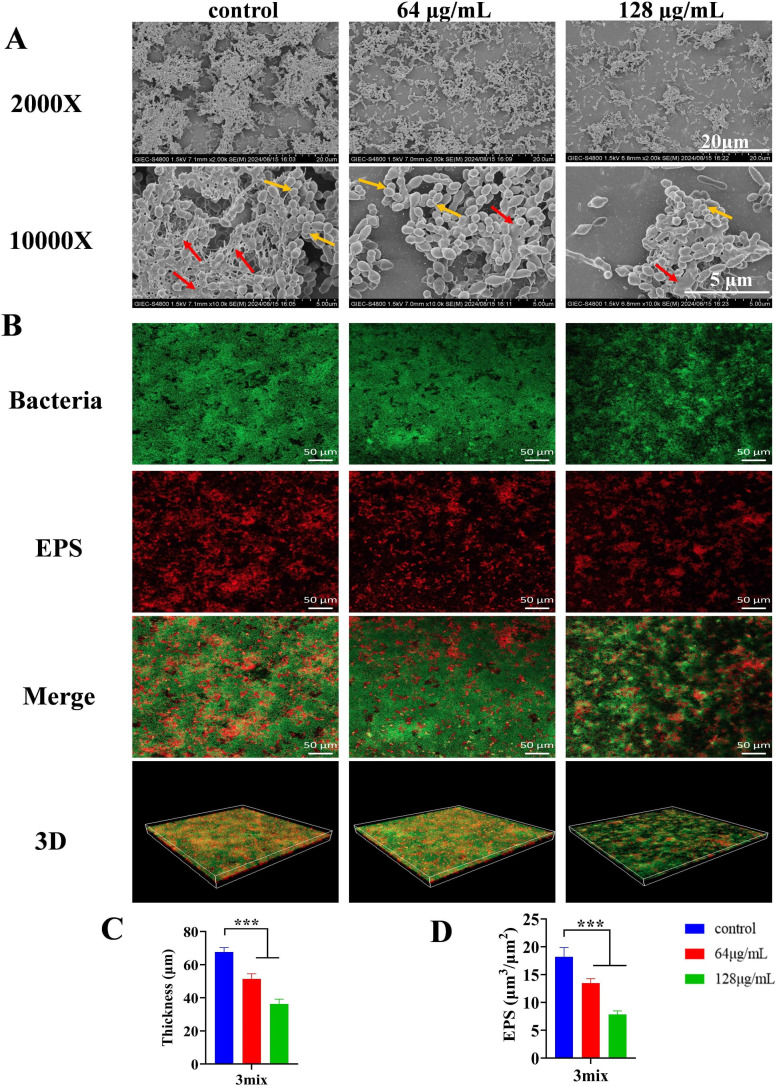
Effect of MUC on multispecies biofilm morphology, thickness, and EPS accumulation. (**A**) Morphology of multispecies biofilms treated with various concentrations of MUC, visualized by SEM at 2,000× and 10,000× magnifications. (**B**) Representative CLSM images (20×) of multispecies biofilms treated with various concentrations of MUC. Green fluorescence (SYTO9) represents bacterial cells, while red fluorescence (dextran labeled with Alexa Fluor 647) represents EPS. (**C**) Quantification of biofilm thickness. (**D**) Quantification of EPS accumulation within biofilms. Red arrows indicate the extracellular matrix; yellow arrows indicate clustered microorganisms. ***P < 0.001.

The morphology of multispecies biofilms treated with MUC was examined using SEM. As the MUC concentration increased, both bacterial density and the extracellular matrix gradually decreased (Fig. 8A). CLSM three-dimensional reconstruction further revealed that MUC significantly decreased biofilm thickness and EPS accumulation in multispecies biofilms (Fig. 8B through D).

### Modulation of bacterial viability in multispecies biofilms by low concentrations of MUC

**Fig 9:**
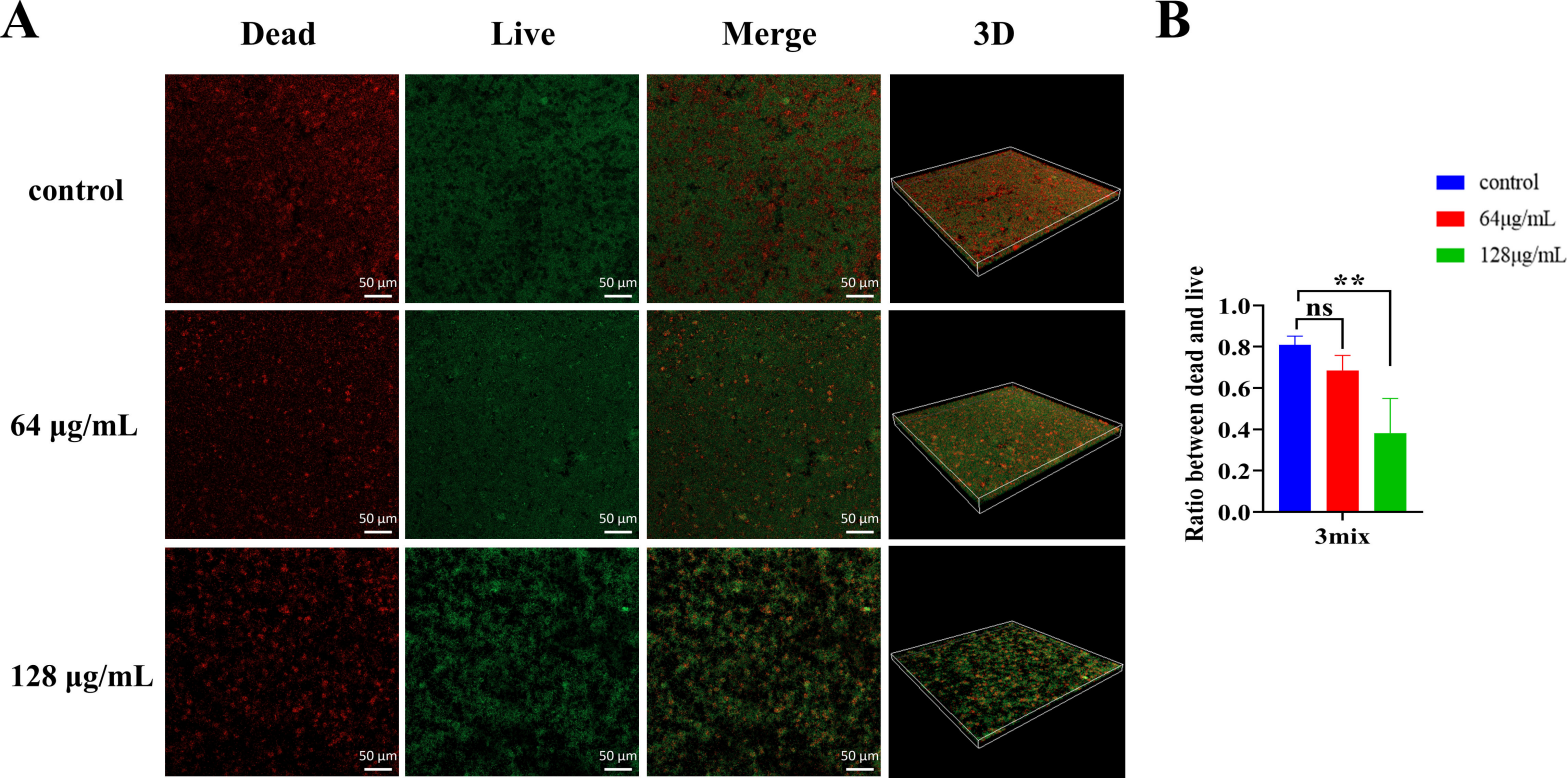
Effect of MUC on bacterial viability in multispecies biofilms. (**A**) Representative CLSM images (20×) of dead and live bacteria within the biofilms. Red fluorescence (PI) indicates dead bacteria, while green fluorescence (SYTO9) indicates live bacteria. (**B**) Ratio of dead to live bacteria within multispecies biofilms. **P < 0.01, ns: no statistical significance.

To further examine the effects of MUC on multispecies biofilms, live/dead staining was conducted (Fig. 9A). The findings indicated a decrease in the ratio of dead to live bacteria within multispecies biofilms comprising *S. mutans*, *S. gordonii*, and *S. sanguinis* at 128 µg/mL (Fig. 9B).

### Reduction of *S. mutans* proportion in multispecies biofilm by streptococcal antagonism promoted by MUC

**Fig 10:**
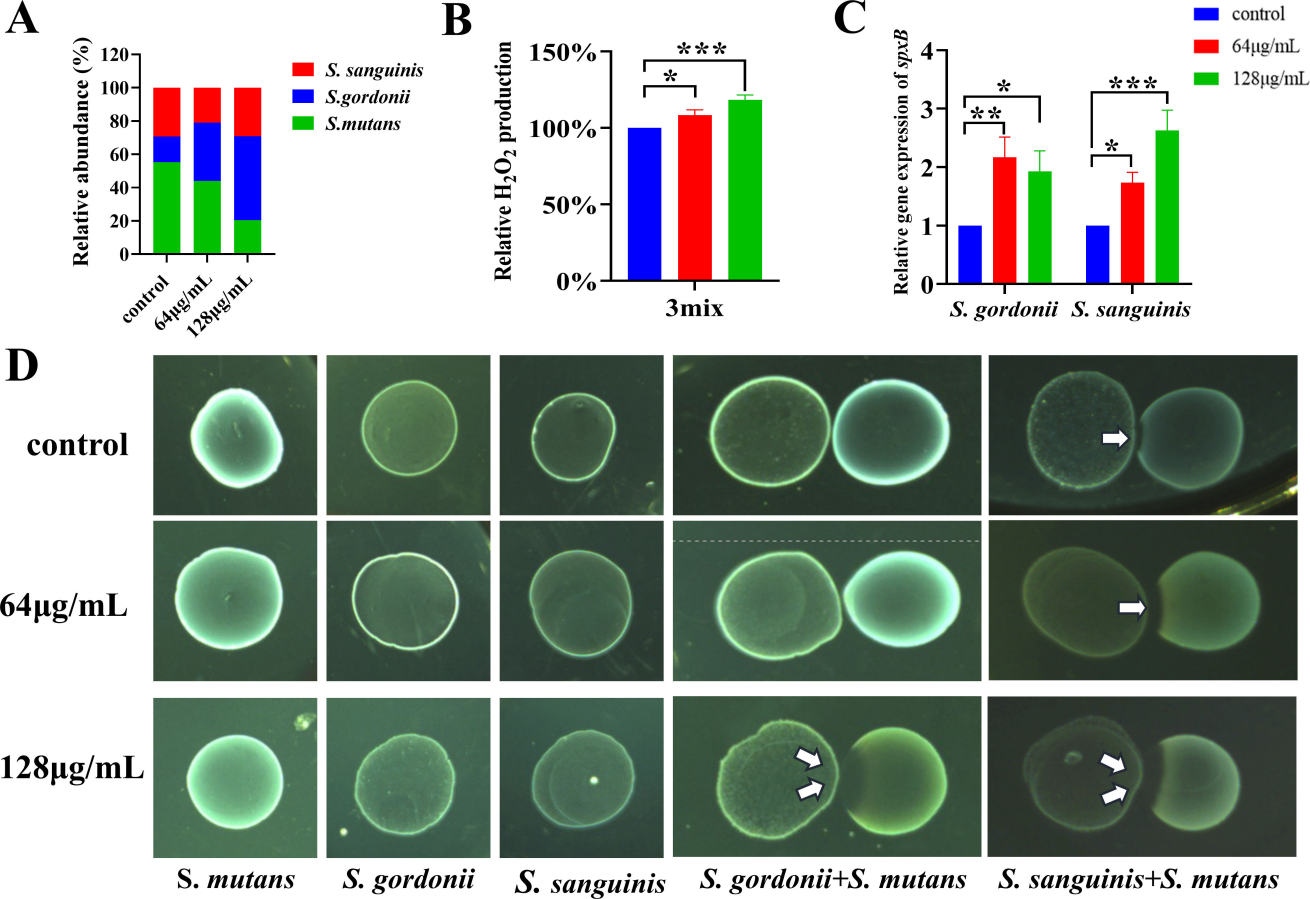
Promotion of streptococcal antagonism by MUC via increased H_2_O_2_ production and upregulated *spxB* expression. (**A**) Proportions of *S. mutans*, *S. gordonii*, and *S. sanguinis* in multispecies biofilms, quantified by TaqMan real-time polymerase chain reaction. (**B**) Relative H_2_O_2_ production in multispecies biofilm treated with MUC for 24 h. (**C**) mRNA expression levels of *spxB* within multispecies biofilms, quantified by qRT-PCR. (**D**) Growth inhibition of *S. mutans* by *S. gordonii* and *S. sanguinis* in the presence of MUC, assessed by an agar plate assay. *P < 0.05, **P < 0.01, ***P < 0.001.

MUC (64 and 128 µg/mL) promoted the planktonic proliferation of *S. gordonii* and *S. sanguinis* while inhibiting that of *S. mutans*. Furthermore, *S. mutans* is the primary species responsible for the production of lactic acid and WIG among oral streptococci. Accordingly, we postulated that a decrease in the percentage of *S. mutans* caused the decrease in lactic acid and WIG in multispecies biofilms treated with MUC. We assessed the bacterial composition of biofilms following 24 h of MUC treatment to evaluate this hypothesis. The analysis indicated that MUC treatment decreased the proportion of *S. mutans* within the multispecies biofilm. Conversely, the percentage of *S. gordonii* and *S. sanguinis* increased following MUC treatment (Fig. 10A).

The results could not be fully explained by the antibacterial properties against *S. mutans* of MUC, as doses of 64 and 128 µg/mL significantly promoted the growth of commensal streptococci. These observations suggested that MUC might influence microbial composition by enhancing antagonistic interactions among streptococcal species. The generation of H_2_O_2_ facilitated by *spxB* in *S. gordonii* and *S. sanguinis* is a well-documented mechanism for suppressing the proliferation of *S. mutans*. To determine whether the growth suppression of *S. mutans* following MUC treatment was due to H_2_O_2_-dependent antagonism, we measured H_2_O_2_ production. The analysis revealed that MUC treatment significantly enhanced H_2_O_2_ production in the multispecies biofilm (Fig. 10B). In accordance with the elevated H_2_O_2_ levels, the expression of *spxB* was significantly upregulated after MUC treatment (Fig. 10C).

We conducted an agar plate assay to further evaluate the antagonistic effects of *S. gordonii* and *S. sanguinis* on *S. mutans*. In the control group, both *S. gordonii* and *S. sanguinis* displayed mild inhibitory effects on *S. mutans*, evidenced by a thinner lawn of *S. mutans* at the intersection without a clearly defined inhibition zone (Fig. 10D). At 64 or 128 µg/mL MUC concentrations, both strains suppressed the growth of *S. mutans*, as reflected by a larger inhibition zone in comparison to the control group (Fig. 10D). These findings confirmed that MUC reduced the proportion of *S. mutans* in multispecies biofilms by promoting streptococcal antagonism, in addition to its direct inhibitory effect on *S. mutans*.

## DISCUSSION

Two primary categories of secondary metabolites have been identified in *S. mutans*: mutacins (9, 16) and PKs/NRPs (17, 18). Mutacins demonstrate broad-spectrum efficacy against gram-positive bacteria, positioning them as promising candidates for the development of innovative antimicrobial therapies, including against dental caries (8, 9). In contrast, three classes of PKs/NRPs—MUC (10), mutanobactin (19, 20), and mutanofactin (13)—have been identified more recently and demonstrate a narrower spectrum of antibacterial activity. Despite their comparatively limited antimicrobial effects, these metabolites play critical roles in promoting virulence, mediating stress responses, providing defense mechanisms, and facilitating biofilm formation. The *BGC1* gene, which encodes MUC, is uniquely found in the genomes of oral streptococci (predominantly *S. mutans*) in humans and primates. This specific genomic distribution indicates that both the gene and its associated metabolite may serve as an adaptive mechanism for survival in the oral environment (10). Consequently, understanding the role of MUC in *S. mutans* and its interactions with other commensal streptococci is important.

Hao et al. (10) reported that MUC has limited antibacterial activity. Similarly, Uranga et al. (14) found that MUC was significantly less effective at inhibiting salivary biofilm formation, requiring concentrations between 100 and 500 µM to achieve a 50% reduction. Consistent with these observations, our study determined that the MIC of MUC against *S. mutans* is 1,024 µg/mL. Furthermore, concentrations exceeding 64 µg/mL were required for MUC to significantly impede biofilm development, as well as the synthesis of WIG and EPS. Microbial communities encased in an extracellular matrix to form biofilms, which are highly structured entities that contribute to several diseases, including dental caries (5). WIG and EPS production directly promote microbial adherence to surfaces and create a polymeric matrix that enhances the structural integrity of biofilms. Moreover, this matrix supports the organization of cells into cohesive multicellular ecosystems, wherein both cooperative and antagonistic interactions occur within a heterogeneous chemical and physical environment, creating localized niches with diverse pathogenic potentials (5). In our study, we employed SEM and CLSM to examine the morphology, three-dimensional structure, and EPS synthesis of biofilms. Our results demonstrated that MUC significantly reduced both the biomass and thickness of the biofilm, as well as the amount of extracellular matrix.

*S. mutans* metabolizes fermentable carbohydrates, resulting in the production of lactic acid as a by-product. The accumulation of lactic acid on the tooth surface decreases pH, resulting in the demineralization of tooth enamel and creating conditions conducive to the development of dental caries. In our study, MUC significantly reduced lactic acid production. Glycolysis serves as the primary pathway for acid production in *S. mutans*. Lactate dehydrogenase (LDH), encoded by the *ldh* gene, is a critical enzyme in this process. Our results demonstrated that MUC suppressed *ldh* gene expression, consistent with observed reductions in lactic acid production. Furthermore, sucrose-dependent adhesion in *S. mutans* primarily involves glucosyltransferases (GTFs) and glucan-binding proteins (GBPs). In a sucrose-rich environment, *S. mutans* synthesizes glucans via GTFs, with glucosyltransferases B (GtfBs) primarily synthesizing insoluble glucans (21). Glucan-binding proteins C (GbpCs), a category of surface-associated proteins, promote bacterial adhesion to glucans and contribute to biofilm development (22). The addition of MUC reduced the expression of *gtfb* and *gbpC* in *S. mutans* biofilms.

The *comDE* system is a well-established two-component system primarily involved in stress responses, such as those to oxygen, acidity, and antibiotics. It also contributes to biofilm development, bacteriocin synthesis, and autophagy (23). In the *vicR*K two-component signaling system, the histidine kinase VicK detects environmental stimuli, transfers phosphorylation signals, and activates the response regulator *vicR*. Upon activation, *vicR* enhances the bacterial traits associated with the organism (24). In our investigation, the addition of MUC significantly increased the expression of *comDE* and *vicR* in *S. mutans* biofilms. Although the upregulation of the *comDE* and *vicRK* systems typically promotes biofilm formation by enhancing cell-to-cell communication and structural stability, our findings demonstrated a significant reduction in EPS accumulation. As a secondary metabolite, MUC may act as an environmental stress signal, triggering *S. mutans* to upregulate *comDE* and *vicR* expression as a defensive response. The ComDE and VicRK systems are essential for *S. mutans* responses to environmental challenges (6, 24). Moreover, MUC treatment may reduce total EPS production in biofilms by suppressing *S. mutans* growth. While *vicR* directly activates the promoter regions of *gtfB* to promote EPS synthesis (25), MUC may inhibit *gtfB* transcription by disrupting its promoter regions. Our data confirmed that MUC treatment significantly reduced *gtfB* expression, which is critical for EPS production. Furthermore, we speculate that MUC may upregulate negative regulators, such as *covR*. Studies have shown that *covR* responds to environmental stress and that *covR*-deficient *S. mutans* is more sensitive to hydrogen peroxide stimulation (26). *covR* directly binds to the promoter regions of *gtfB,* suppressing its transcription, and also represses the expression of *gbpC* (25, 27). Zhang et al. reported that *covR* may exert dominant regulatory control through interactions with the VicR pathway (25, 28). Our findings and speculation highlight the complexity of *S. mutans* responses to pharmacological stress and suggest that MUC may employ a multifaceted mechanism of action. Further experiments are required to elucidate these potential regulatory pathways.

Our findings also demonstrated that MUC, a secondary metabolite of *S. mutans*, inhibited *S. mutans* growth while promoting the growth of *S. gordonii* and *S. sanguinis*, highlighting a complex ecological interaction. Zhang et al. (29) found that *Limosilactobacillus* species produce reutericyclin (RTC), which exhibits potent antibacterial activity and suppresses *S. mutans* growth (12, 30). However, *S. mutans* can convert RTC to MUC. By producing MUC, *S. mutans* reduces its own cariogenic potential to some extent, while simultaneously enhancing the survival opportunity. Moreover, the promotion of *S. gordonii* and *S. sanguinis* by MUC is consistent with the concept of “public goods” in microbial ecology, where metabolites are produced at a cost to the producer to benefit neighboring species (31).

Our study used a well-established multispecies biofilm model to evaluate the anticaries potential of MUC (32). *S. mutans* is the most significant cariogenic bacterium among these species, whereas *S. gordonii* and *S. sanguinis* are typically considered harmless commensals concerning caries development. Compared to monospecies biofilms of *S. mutans*, MUC exhibited a more substantial inhibitory effect on the cariogenicity of multispecies biofilms, specifically by reducing biofilm formation, lactic acid production, and the synthesis of WIG and EPS. Increasing concern exists regarding the effects of antimicrobials on oral microbial communities. Broad-spectrum antimicrobials disrupt the interactions between pathogenic and commensal microorganisms, leading to dysbiosis. Our results indicated that MUC reduced the proportion of *S. mutans* in multispecies biofilms by promoting interspecies antagonism, as evidenced by increased H_2_O_2_ production and upregulation of the *spxB* gene in *S. gordonii* and *S. sanguinis*. *SpxB* encodes pyruvate oxidase, which catalyzes the conversion of pyruvate to H_2_O_2_, enabling *S. gordonii* and *S. sanguinis* to gain a competitive advantage when co-cultured with *S. mutans* (33, 34). In *S. sanguinis*, *spxA1* is strongly associated with environmental stress responses and can induce *spxB* expression (35). As a secondary metabolite, we speculate that MUC may act as an environmental stress signal, prompting *S. sanguinis* to upregulate *spxA1* expression, which in turn promotes *spxB* transcription. Further experimental validation is required to substantiate this hypothesis. Furthermore, other studies have also reported a decrease in the proportion of *S. mutans* in multispecies biofilms following antimicrobial treatment (35, 36). These findings highlight the potential of MUC as an "ecological regulator" in caries prevention, specifically by enhancing the antagonism of *S. gordonii* and *S. sanguinis* against *S. mutans*.

It is important to note that the effects of MUC are observed at concentrations of at least 64 µg/mL, which is relatively high and may not be physiologically relevant to the concentrations typically produced by *S. mutans* in a multispecies biofilm. However, future applications of gene editing could be used to promote *BGC1* gene expression in *S. mutans*, thereby enhancing the production of MUC within the cariogenic microenvironment. This approach could reduce the virulence of *S. mutans*, *C. albicans,* and *L. fermentum*, while fostering the proliferation of beneficial symbiotic bacteria such as *S. gordonii* and *S. sanguinis*.

Generally, antimicrobial treatments increase the ratio of dead to live bacteria in biofilms. In contrast, our study found that MUC reduced the dead-to-live ratio within multispecies biofilms. However, MUC increased the ratio of live to dead bacteria in *S. mutans* biofilms in a dose-dependent manner. We hypothesize that, in the multispecies biofilm, MUC (64 and 128 µg/mL) promoted the proliferation of *S. gordonii* and *S. sanguinis* while increasing the proportion of dead *S. mutans*. This shift likely contributed to a lower dead-to-live bacterial ratio in the CLSM quantitative analysis. These findings supported the hypothesis that MUC may promote the development of a healthier multispecies biofilm.

## MATERIALS AND METHODS

### Bacterial strains and chemicals

*S. mutans* UA159 (ATCC 700610), *S. sanguinis* (ATCC 10556), and *S. gordonii* DL-1 were sourced from our laboratory. These bacterial strains had previously undergone sequencing and comparative analysis. Cultivation was conducted in brain heart infusion (BHI) medium (Difco, Detroit, MI, USA) at 37°C. The Intelligent Anaerobic Microbial Cultivation System (DW-100A-K Series, Hangzhou Dawei Biotechnology Co., China) was used to produce microaerophilic conditions, with a mixed gas composition containing 6% oxygen, 3.6% H_2_, 3.6% CO_2_, and 86.8% N_2_.

Bacterial concentrations were standardized to 1 × 10^6^ CFU/mL for subsequent experiments. Individual bacterial strains were injected into BHI medium enriched with 1% (wt/vol) sucrose (BHIS) for monospecies biofilm development. Bacterial suspensions were combined to create inocula consisting of a microbial consortium: *S. mutans*, *S. gordonii*, and *S. sanguinis*, referred to as “3mix” for the multispecies biofilm. MUC powder, sourced from Bide Pharmatech (Guangzhou, China), was dissolved in methanol to prepare stock solutions at various concentrations. The solutions were formulated to maintain methanol at 0.5% of the total liquid volume for subsequent experiments and were stored at 4°C until required for use.

### Hemolytic assay

Fresh rabbit blood (Cat.E0503; Pingrui Biotech, Beijing, China) was washed with phosphate-buffered saline (PBS) in a centrifuge tube to eliminate fibrous proteins. Subsequently, centrifugation (1,500 rpm) was performed for 5 min, resulting in the concentration of blood cells in the pellet. This step was repeated five times. The blood cells were then mixed with different concentrations of MUC, with distilled water serving as the positive control. For the negative control, blood cells were combined with PBS. The samples were incubated at 37°C for 1 h, followed by centrifugation for 5 min (1,500 rpm). The OD of the supernatant was quantified at 540 nm (OD_540_) by microplate reader (Tecan, Reading, Switzerland). The OD_540_ values were then used to perform statistical analysis to quantify the results of the hemolysis assay.

### Cell cytotoxicity test

HOK cells were obtained from our laboratory. Cells were cultured in Dulbecco’s modified Eagle medium (DMEM; Gibco, MA, USA) and seeded in 96-well plates at an initial density of 1 × 10^4^ cells/well, followed by 24 h incubation at 37°C. The culture medium was subsequently replaced with fresh medium containing various concentrations of MUC. For the control, cells were cultured without MUC. After an additional 24 h incubation, the medium was removed. Each well received 10 µL of Cell Counting Kit-8 (CCK-8; Dojindo, Kumamoto, Japan) solution combined with 100 µL of fresh medium and incubated for 2 h. The absorbance was measured at 450 nm (OD_450_) using a microplate reader. Cytotoxicity was assessed by calculating relative cell viability, expressed as a percentage of the untreated control group, using the following formula: Cell viability (%) = {[A(treatment) − A(blank)]/[A(control) − A(blank)]} × 100. Here, A(treatment) represents the OD_450_ of wells containing cells, CCK-8 solution, and MUC; A(control) represents the OD_450_ of wells with cells and CCK-8 solution but without MUC; and A(blank) represents the OD_450_ of wells without cells.

### MIC test of MUC

The Clinical and Laboratory Standards Institute (CLSI) experimental methods were employed to determine the MIC of MUC against *S. mutans*, *S. gordonii*, and *S. sanguinis* through serial microdilution assays. Briefly, the initial concentration of microorganisms was standardized to 1 × 10^6^ CFUs/mL. The MIC was established as the minimal concentration of MUC, which entirely suppressed bacterial growth relative to the control group, as evidenced by the absence of visible bacterial growth in the wells after 24 h. The control group was treated with a 0.5% (vol/vol) methanol solution.

### Planktonic growth assays

Bacterial cultures were incubated overnight in BHI broth with various concentrations of MUC. The control group was supplemented with BHI containing 0.5% (vol/vol) methanol solution but without MUC. Planktonic microbial proliferation was quantified by a microplate reader at 600 nm (OD_600_) every 2 h. The OD_600_ values recorded at 22 h were subsequently subjected to statistical analysis. Simultaneously, we applied methanol solutions of various concentrations to *S. mutans* to determine whether 0.5% (vol/vol) methanol is a suitable solvent for MUC in subsequent experiments.

### Biofilm formation assays

The concentration of microorganisms was standardized to 1 × 10^6^ CFUs/mL. Bacteria with various concentrations of MUC in 1% (wt/vol) BHIS were inoculated in 48-well plates for a duration of 24 h under microaerophilic conditions (6% O_2_). The control group was supplemented with BHIS containing 0.5% (vol/vol) methanol solution but without MUC. The medium and unattached bacteria were eliminated, and PBS was utilized to rinse the wells. After adding methanol for 10 min to fix the biofilms, the supernatant was disposed of, and the biofilms were allowed to dry for 30 min. After 15 min of treatment with a 0.1% (wt/vol) crystal violet solution, any unattached dye in the biofilms was then rinsed off with running water. And the dye incorporated into the biofilms was solubilized in 95% ethanol with gently shaking for 30 min. Using a microplate reader, the absorbance of the dissolved crystal violet was measured at 595 nm.

### CFU counts

Viable bacterial counts in planktonic bacteria and biofilms were determined using CFU counting. For planktonic bacteria, cultures were subjected to serial dilution after 24 h of incubation with or without MUC. After plating the diluted samples on BHI agar, they were incubated microaerophilically for 24 h and CFU counts were calculated based on the resulting colonies. For biofilms, the culture medium and unattached bacteria were discarded, followed by two washes with PBS. After being collected using a cell scraper, the biofilms were vortexed in PBS for 30 s. Subsequently, they were subjected to sonication at a frequency of 40 kHz using an ultrasonic cleaner (Boxun, China) for 5 min to ensure complete resuspension, followed by serial dilution to quantify CFU.

### Quantitative determination of WIG

The analysis of WIG was conducted utilizing a modified phenol-H_2_SO_4_ method (37). Briefly, biofilms in the presence of MUC were grown in 24-well plates for 24 h. The control group was supplemented with BHIS containing 0.5% (vol/vol) methanol solution but without MUC. The biofilms underwent gentle rinsing with PBS two times, followed by resuspension and 10 min centrifugation (4,000 × *g*). After that, the precipitate was reconstituted in 0.1 M NaOH and brought to 37°C for 2 h of incubation. The EPS was precipitated by filtering the supernatant through 0.22 µm nitrocellulose membrane filters (Cat. BS-PTFE25-22-L, Biosharp, Hefei, China) after a 10 min centrifugation. The mixture was incubated overnight after three volumes of cold 95% ethanol were added. One volume of 5% (wt/vol) ice-cold phenol and five volumes of concentrated H_2_SO_4_ were combined with one volume of supernatant. Finally, the absorbance of each well was measured using a microplate reader at 492 nm.

### Lactic acid measurement

Biofilms were cultivated in 48-well plates according to previously established methods. The biofilms were washed with PBS and incubated with buffered peptone water (BPW; Solarbio, Beijing, China) containing 0.2% (wt/vol) sucrose in each well. The biofilms were incubated microaerophilicly at 37°C for 2 h. Afterward, the supernatants were analyzed utilizing the lactate assay kit (Cat. A019-2; Jiancheng, Nanjing, China). Following the manufacturer’s instructions, the absorbance was measured at 530 nm and the lactic acid concentration was calculated.

### SEM of biofilms

SEM (ZEISS, Oberkochen, Germany) was utilized to visualize the structural alterations in biofilms following MUC treatment. 14 mm cell slides (WHB, China) are pre-placed in six-well plates. Following removal of the medium, the biofilms underwent three PBS rinses before being fixed for 6 h with 2.5% (wt/vol) glutaraldehyde. Subsequently, the biofilms were rinsed with PBS and dehydrated with ethanol solutions of increasing concentrations (50%, 70%, 80%, 90%, and 100%), with each concentration applied for 15 min. The samples underwent treatment with tert-butanol solution three times (15 min per treatment) and were subsequently lyophilized overnight. The biofilms on cell slides were sputter-coated with gold and imaged using SEM at magnifications of 2,000× and 10,000×.

### CLSM of biofilms

According to the preceding description, biofilms were cultivated on 15 mm confocal dishes with MUC. Untreated biofilms served as the negative control. CLSM (Zeiss LSM 980, Oberkochen, Germany) was utilized for live/dead cell and EPS imaging. Prior to biofilm formation, each plate was treated with dextran conjugate (2.5 µM) labeled with Alexa Fluor 647 (Invitrogen, CA, USA) for EPS staining. The biofilms were washed with PBS following the disposal of the supernatant after a 24 h cultivation period. Next, the biofilms were treated with 2.5 µM SYTO9 dye (Invitrogen, CA, USA) in a dark environment lasting 15 min. SYTO9 exhibits excitation and emission maxima of 480 and 500 nm, respectively. The dextran conjugate labeled with Alexa Fluor 647 shows excitation and emission maxima of 652 and 668 nm, respectively. We employed COMSTAT 2.1 software to separately quantify the biomass of EPS and bacteria and subsequently calculated the ratio of EPS to bacterial biomass. The thickness of the biofilms was assessed through Z-section measurements. Each experiment involved the analysis of a minimum of five random fields.

For the live/dead staining experiment, the supernatant was discarded, and the biofilms were rinsed with PBS three times in preparation. Following the instructions of the LIVE/DEAD BacLight Bacterial Viability Kit (L7012, Invitrogen, CA, USA), the biofilms were stained in the dark environment for 15 min using an equal-volume mixture of SYTO9 and PI (Invitrogen, MA, USA). For PI, the excitation wavelength was 490 and the emission wavelength was 635 nm. Biofilms were then monitored and imaged by CLSM at 20× magnification. We employed COMSTAT 2.1 software to separately quantify the biomass of dead and live bacteria and subsequently calculated the ratio of dead cells to live cells. The thickness of the biofilms was assessed through Z-section measurements. Each experiment involved the analysis of a minimum of five random fields.

### RNA extraction and qRT-PCR

Biofilms were cultured in the presence of MUC in six-well plates, following the previously described protocol. After incubation for 24 h, biofilms were collected using centrifugation (12,000 rpm) for 5 min. Total RNA was harvested using the miRNeasy Mini Kit (QIAGEN GmbH, Hilden, Germany). Subsequently, RNA was reverse-transcribed to cDNA using the PrimeScript RT reagent kit (Takara Bio Inc., Otsu, Japan). qRT-PCR was used to measure the relative mRNA expression levels with the Bio-Rad CFX Connect Real-Time PCR System (Bio-Rad, Hercules, CA, USA). The same amount of cDNA was added as template to each testing sample. Primers are listed in Table S1. The 2^−ΔΔCt^ method was employed to normalize gene expression relative to the reference gene (16S rRNA). Specifically, the qRT-PCR instrument automatically generated the cycle threshold (Ct) value for each sample. The ΔCt value was calculated by subtracting the Ct value of the reference gene from that of the target gene. The ΔΔCt value was determined by subtracting the ΔCt of the control group from the ΔCt of the experimental group. The fold change in target gene expression in the experimental group relative to the control group was then quantified using the formula 2^−ΔΔCt^.

The bacterial composition was further quantified using the method described in previous studies (27). The quantities of *S. mutans*, *S. gordonii*, and *S. sanguinis* were measured using TaqMan quantitative real-time PCR (qPCR) with Premix Ex Taq (Probe qPCR). For each primer/probe set, standard curves for these bacteria were created, and the probes' specificity was verified using conventional PCR. To determine the relevant bacterial concentrations, these curves were created by amplifying serial dilutions of 10-fold of DNA with established concentrations, ranging from 10^4^ to 10^8^ CFUs. The quantity of each strain was determined using standard curves generated from the corresponding standard strains. Primers and probes are listed in Table S2.

### H_2_O_2_ measurements

Following the manufacturer’s instructions, a hydrogen peroxide assay kit (Beyotime, Shanghai, China) was employed to quantify H_2_O_2_ production in the biofilm supernatant. After MUC treatment, a transparent 96-well plate was filled with the biofilm supernatant (50 µL) and detection reagent (100 µL). The production of H_2_O_2_ was evaluated by measuring the absorbance at 560 nm following a 30 min incubation at 25°C.

### Competition assays on agar plate

A modified competition assay protocol, as described previously (29), was utilized to assess the inhibitory effects of *S. gordonii* and *S. sanguinis* on *S. mutans*. Briefly, BHI agar plates were inoculated with 10 µL of *S. gordonii* or *S. sanguinis* (OD_600_ = 0.4) as the pioneer colonizer. Following a 12 h incubation, 10 µL of *S. mutans* (OD_600_ = 0.4) was inoculated in proximity to the pioneer colonizer, ensuring that the colonies were in close proximity. The BHI agar plates underwent incubation for an additional 12 h. The presence of an inhibition zone at the colony intersection indicated growth inhibition.

### Statistical analysis

All experiments were conducted in triplicate independently. GraphPad Prism 8 (San Diego, CA, USA) was used for statistical analysis of the data. One-way analysis of variance (ANOVA) was conducted, followed by Dunnett’s or Tukey’s multiple-comparison test.

### Conclusion

In this study, we assessed the effects of MUC on *S. mutans* and other oral commensal streptococci in both mono- and multispecies biofilms. Our findings demonstrated that MUC (exceeding 64 µg/mL) exhibited low cytotoxicity and displayed potent antibacterial activity against *S. mutans*. MUC exhibited a significantly greater inhibitory effect on cariogenicity in multispecies biofilms than in monospecies *S. mutans* biofilms. Additionally, MUC reduced the abundance of *S. mutans* and enhanced the antagonistic activity of *S. gordonii* and *S. sanguinis* against *S. mutans* by upregulating the expression of the H_2_O_2_-related gene *spxB* and stimulating H_2_O_2_ production. These data suggest that MUC has potential anti-cariogenic activity against *S. mutans* and can modulate oral microbial communities within the cariogenic microenvironment, offering potential new therapeutic strategies for the prevention of dental caries.

## SUPPLEMENTAL MATERIAL

Figure S1, and Tables S1 and S2 spectrum.00183-25-s0001.pdf Figure S1: Growth curves of S. mutans treated with various concentrations of methanol. Table S1: Primers used for real-time quantitative polymerase chain reaction. Table S2: Primers and probes used for species-specific real-time quantitative polymerase chain reaction.

ASM does not own the copyrights to Supplemental Material that may be linked to, or accessed through, an article. The authors have granted ASM a non-exclusive, world-wide license to publish the Supplemental Material files. Please contact the corresponding author directly for reuse.
