# Supplementary material for: Anti-cariogenic activity of mutanocyclin, a secondary metabolite of Streptococcus mutans, in mono- and multispecies biofilms
Source: Microbiol Spectr. 2025 Jun 25;13(8):e00183-25. doi: 10.1128/spectrum.00183-25 (PMC12323323; doi:10.1128/spectrum.00183-25)
Supplement: Figure S1, and Tables S1 and S2 — Figure S1: Growth curves of S. mutans treated with various concentrations of methanol. Table S1: Primers used for real-time quantitative polymerase chain reaction. Table S2: Primers and probes used for species-specific real-time quantitative polymerase chain reaction. [file spectrum.00183-25-s0001.pdf]

Supplementary materials

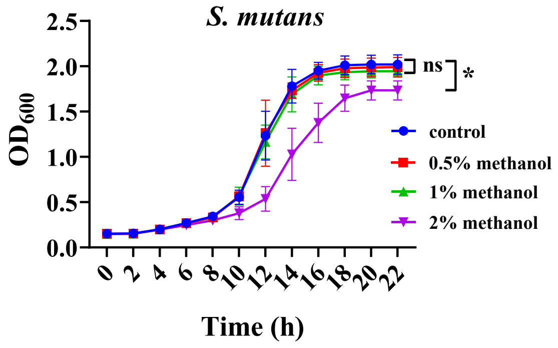

Supplementary Figure S1. Growth curves of *S. mutans* treated with various concentrations of methanol. *$P < 0.05$, ns: no statistical significance.

Supplementary Table S1. Primers used for real-time quantitative polymerase chain reaction.

Genes	Primer Sequence (Forward and Reverse)
16s rRNA *S. mutans*	F: CGGCAAGCTAATCTCTGAAA
	R: GCCCCTAAAAGGTTACCTC
16S rRNA *S. gordonii*	F: GCTTGCTACACCATAGACTG
	R: TCCTCATCACCATCCATAAAG
16S rRNA *S. sanguinis*	F: AAGCAACGCGAAGAACCTTA
	R: GTCTCGCTAGAGTGCCCAAC
gtfB	F: AGCCGAAAGTTGGTATCGTCC
	R: TGACGCTGTGTTTCTTGGCTC
gbpC	F: TCTGGTTTTTCTGGCGGTGT
	R: GTCAATGCTGATGGAACGCC
ldh	F: AAAAACCAGGCGAAACTCGC
	R: CTGAACGCGCATCAACATCA
comDE	F: ACAATTCCTTGAGTTCCATCCAAG
	R: TGGTCTGCTGCCTGTTGC
vicK	F: CGTGTAAAAGCGCATCTTCG
	R: AATGTTCACGCGTCATCACC

spxB S. gordonii	F: TTGCAGTAGGTTCAGGTGGT
	R: GGCAAGCTTCGTCAATCACT
spxB S. sanguinis	F: AATTCGGCGGCTCAATCG
	R: AAGGATAGCAAGGAATGGAGTG

Supplementary Table S2. Primers and probes used for species-specific real-time quantitative polymerase chain reaction.

Primers/Probes	Primer Sequence (5′–3′)
Primers:	
S. mutans	F: GCCTACAGCTCAGAGATGCTATTC
	R: GCCATACACCACTCATGAATTGA
S. gordonii	F: GGTGTTGTTTGACCCGTTCAG
	R: AGTCCATCCCACGAGCACAG
S. sanguinis	F: GAGCGGATGGCCAATTATATCT
	R: CCGGATGATGTCGGCAATA
Probes:	
S. mutans	FAM-TGGAAATGACGGTCGCCGTTATGAA-TAMRA
S. gordonii	FAM-AACCTTGACCCGCTCATTACCAGCTAGTATG- TAMRA
S. sanguinis	FAM-TGTTCGGGCTCATGATA-Eclipse
